# Ontario Veterinary Medical Society

**Published:** 1894-02

**Authors:** J. S. Dade

**Affiliations:** Secretary


### ONTARIO VETERINARY MEDICAL SOCIETY.

The following is a brief monthly report of the papers read and discussed in the Ontario Veterinary Medical Society which meets semi-weekly and has for its officers: President, Prof. Smith; Vice-President, Prof. Sweetapple; Secretary, J. S. Dade; Assistant Secretary, E. G. Britton; Treasurer, Judson Black; Librarian, J. McGillicuddy. The following were among those responding with papers:

1. L. R. Webber, "Glanders"; 2. E. M. Nighbert, "Symptomatic Anthrax"; 3. W. N. Armstrong, "Azoturia"; 4. M. B. Rombough, "Cerebro-spinal Meningitis"; 5. J. McGuffin, "Apoplexy"; 6. W. R. Fullerton, "Open Joint"; 7. J. M. Douglas, "Purpura"; 8. W. R. Bell, "Principles and Practice of Horse-shoeing"; 9. R. N. Morgan, "Heredity"; 10. R. Colthurst, "Roaring"; 11. W. Kreider, "Eccentric Hypertrophy of Heart"; 12. A. W. Baker, "Examination of Horses as to Soundness"; 13. J. Crail, "Lymphangitis"; 14. C. F. Ebner, "Selection of Stock"; 15. A. B. Campbell, "Strangles and its Results"; 16. J. E. Robertson, "Tuberculosis"; 17. R. Turnbull, "Dentigerous Cysts"; 18. C. Nockolds, "The Broncho and his Diseases"; 19. Thos. Flood, "Canine Distemper"; 20. J. E. Robertson, "Irregular Strangles"; 21. C. A. Turley, "Actinomycosis"; 22. A. Barradell, "Tetanus"; 23. L. P. Beechy, "Chronic Spinitis"; 24. W. J. Price, "Acute Rheumatism"; 25. A. W. Whitehouse, "Paris Congress on Tuberculosis"; 26. J. Cunningham, "Chorea"; 27. W. H. Smith, "Hygiene of Animals"; 28. A. J. Kline, "Azoturia"; 29. J. M. Douglas, "Burns and Mechanical Bronchitis"; 30. J. Butler, "Periodic Ophthalmia." There are many that I have omitted in making out this list, but these will suffice to give the reader an idea of the work we are pursuing.

The merit of these papers was such as to demand lengthy and complete discussion—the discussions are always carried on with spirit, the writer being expected to defend his paper against all attacks.

From the list of papers given above, some idea may be formed of the activity which characterizes the work of this Society. Under the guiding hand of the President, each one strives to do his part well, thus laying the foundation for success in the future—after he has finally left the walls of his alma mater—and entered the ranks of the veterinary profession.

J. S. Dade, *Secretary*.

---

### NOTICE.

Additional copies of the Proceedings of the United States Veterinary Medical Association for 1891 and 1892, having been secured by the officers, may be obtained at the Secretary's office, for the cost price of $2.00 per cloth bound, and $1.75 per paper copy.

Subscription membership is still open at $2.00 for the Proceedings of the Thirtieth Annual Meeting and the First International Congress.

Columbia, Mo. T. J. Turner, *Secretary*.

